# *Cannabis sativa* subsp. *sativa*’s pharmacological properties and health effects: A scoping review of current evidence

**DOI:** 10.1371/journal.pone.0245471

**Published:** 2021-01-19

**Authors:** Xin Yi Lim, Terence Yew Chin Tan, Siti Hajar Muhd Rosli, Muhammad Nor Farhan Sa’at, Syazwani Sirdar Ali, Ami Fazlin Syed Mohamed

**Affiliations:** Herbal Medicine Research Centre, Institute for Medical Research, National Institutes of Health, Ministry of Health Malaysia, Setia Alam, Shah Alam, Malaysia; Bhagwan Mahvir College of Pharmacy, INDIA

## Abstract

**Introduction:**

Hemp (*Cannabis sativa* subsp. *sativa*), commonly used for industrial purposes, is now being consumed by the public for various health promoting effects. As popularity of hemp research and claims of beneficial effects rises, a systematic collection of current scientific evidence on hemp’s health effects and pharmacological properties is needed to guide future research, clinical, and policy decision making.

**Objective:**

To provide an overview and identify the present landscape of hemp research topics, trends, and gaps.

**Methods:**

A systematic search and analysis strategy according to the preferred reporting items for systematic review and meta-analysis-ScR (PRISMA-ScR) checklist on electronic databases including MEDLINE, OVID (OVFT, APC Journal Club, EBM Reviews), Cochrane Library Central and Clinicaltrials.gov was conducted to include and analyse hemp research articles from 2009 to 2019.

**Results:**

65 primary articles (18 clinical, 47 pre-clinical) were reviewed. Several randomised controlled trials showed hempseed pills (in Traditional Chinese Medicine formulation MaZiRenWan) improving spontaneous bowel movement in functional constipation. There was also evidence suggesting benefits in cannabis dependence, epilepsy, and anxiety disorders. Pre-clinically, hemp derivatives showed potential anti-oxidative, anti-hypertensive, anti-inflammatory, anti-diabetic, anti-neuroinflammatory, anti-arthritic, anti-acne, and anti-microbial activities. Renal protective effects and estrogenic properties were also exhibited *in vitro*.

**Conclusion:**

Current evidence on hemp-specific interventions are still preliminary, with limited high quality clinical evidence for any specific therapeutic indication. This is mainly due to the wide variation in test item formulation, as the multiple variants of this plant differ in their phytochemical and bioactive compounds. Future empirical research should focus on standardising the hemp plant for pharmaceutical use, and uniformity in experimental designs to strengthen the premise of using hemp in medicine.

## Introduction

Hemp and marijuana belong to the same *Cannabis sativa* plant species, differing botanically at a subspecies level as *C*. *sativa* subsp. *sativa* (hemp) and *C*. *sativa* subsp. *indica* (marijuana) [1]. While marijuana has long gained popularity for medical uses [2], the rise of hemp research is relatively recent, as it is traditionally used for industrial purposes [3–7]. Legally, hemp is distinguished from marijuana according to the allowable upper limit of the psychoactive delta-9-Tetrahydrocannabidiol (THC). The European Union caps the levels of delta-9-THC in hemp at 0.2% [8] while the U.S Agricultural Marketing Act 1946 restricts delta-9-THC to less than 0.3% on a dry weight basis [9]. Current U.S Food and Drug Administration approved cannabis-based drugs for therapeutic use are either synthetic or derived from the medical marijuana, not hemp, indicating that evidence supporting use of hemp for therapeutic purposes may still be insufficient [10–12].

Due to its non-psychoactive nature, nutrient-rich content, and easy accessibility in several countries, hemp has increasingly garnered public attention and is being commercialised and consumed by the general public as health promoting and benefiting products, with or without cannabidiol (CBD), another major cannabinoid found in the Cannabis plant [13–15]. Recently, there is emerging evidence of non-cannabinoid active compounds contributing towards pharmacological effects of hemp [16, 17]. This evidence is different from current published systematic reviews of Cannabis, which have focused mainly on cannabinoids [18, 19]. A narrative review by Crescente et al., 2018 highlighted the historical use, phytochemicals, and some potential nutraceutical properties of hemp. However, there was a lack of systematic research, compilation, and analysis of all related studies and therefore this review does not provide an objective overview of current hemp research [14].

Despite hemp’s growing popularity, systematic collection of available scientific evidence detailing the beneficial health effects in humans and pharmacological properties specific to hemp is limited. Since hemp related biomedical research is a relatively new field of study, such evidence syntheses are valuable in guiding future research, clinical recommendations, and policy decisions making [20]. Hence, this paper aims to delineate the current landscape of hemp research, through recent scientific findings specific to the pharmacological properties of the hemp plant and its derived compounds, at both pre-clinical and clinical levels. Here, we present an overview of the research characteristics, patterns, and trends, identify gaps, and suggest a focus point for future research for the hemp plant. To our knowledge, this is the first scoping review on hemp’s overall pharmacological properties and health effects.

## Materials and methods

This scoping review was conducted based on the York framework as outlined by Arksey and O’Malley [21] and Levac *et al*. [22]. The five stages of scoping review underttaken include (1) identifying research question(s), (2) identifying studies relevant to research question(s), (3) selecting studies, (4) extracting and charting data, and (5) collating, summarising, and presenting data. This scoping review protocol is not registered with PROSPERO as it included both human and animal studies and therefore did not meet the inclusion criteria of accepted protocol registration.

### Stage 1: Identifying research question

The main research question addressed was “What is the current scientific evidence available on hemp’s pharmacological properties and health effects?” while the secondary questions are “What are the hemp research trends, gaps and challenges?” As this review aimed to present all the evidence related and specific only to hemp, the Population, Concept, and Context (PCC) framework was applied for a more flexible approach instead of the Population, Intervention, Comparison and Outcomes (PICO) framework **(**Table 1**)**.

**Table 1:** Population, Concept, and Context (PCC) elements of research question.

**Elements**	**Details**
Population	Hemp/ hemp derived compound/hemp derived product “users” in all types of study models. “Users” in this context is defined as any system i.e. assays, *in vitro* (e.g. cell lines), *in vivo* (animals) or human (including case reports and series in human studies) which have been administered hemp or hemp derived products or compounds for research purposes.
	All human study population, patients, and healthy volunteers, at all age levels.
Concept or Intervention	Hemp/ hemp derived compound/hemp derived product, of any formulation, either as individual formulation or in any mixture.
	All studies utilising the hemp plant, regardless of part of plant use e.g. seed, flowering top, stalk etc.
Context or Outcome	Pharmacological properties in preclinical studies which can potentially be used in furthering research of the product for therapeutic purposes.
	Health effects as defined by any therapeutic or physiological effects (positive, negative, and no effects) reported in clinical studies.

### Stage 2 and 3: Identifying and selecting relevant studies

#### Search strategy

A systematic search on published literature from several electronic databases including MEDLINE, OVID (OVFT, APC Journal Club, EBM Reviews), Cochrane Library Central and Clinicaltrials.gov was conducted by a multidisciplinary team of medical doctors, pharmacists, and a biomedical researcher for English articles from January 2009 to December 2019. The keyword combination applied for MEDLINE, OVID, Cochrane Library Central searches was “Medicinal OR Therapeutic OR Benefit OR Effect OR Properties OR Bioactive” AND “Hemp OR Hemps OR Cannabidiol”. For search on Clinicaltrials.gov, the keyword “Hemp” was used (S1 Appendix). All searches were performed by two independent investigators. A bibliographic software (EndNote X8.1) was used to manage and remove duplicates. For clinical trials registered with clinicaltrials.gov with pending research status and those completed but without published results, attempts were made to contact the lead-investigator to confirm the status and results.

#### Study selection

Articles were selected based on the inclusion criteria and exclusion criteria **(**Table 2**)** expanded from the PCC of research question **(**Table 1**)**, specific to hemp. After title and abstract screening, articles were subjected to full text review by two independent investigators. Any conflicting disparities among both investigators were reviewed by a third investigator.

**Table 2:** Inclusion and exclusion criteria.

*Inclusion criteria*	a) Peer reviewed primary articles b) Articles using hemp as defined as *Cannabis sativa* subsp. *sativa* with less than <0.3% THC or similar as the study intervention c) Articles using hemp derived products or bioactive compounds to evaluate their potential therapeutic or biological effects d) Articles that evaluate hemp, hemp derived products or bioactive compounds in all types of formulations including as raw plant, crude, extracts, single, or combination of extracted bioactive compounds, prepared products as single or mixture of herbs that include hemp
*Exclusion criteria*	a) Articles that use cannabis-based products or compounds in which its origin is marijuana/ hashish/ or formulation with >0.3% THC b) Articles that use cannabis-based products or compounds with unclear origin (i.e., unspecified for hemp or unable to distinguish its plant subspecies origin) c) Articles that evaluate the biological or therapeutic effects of delta-9-THC solely

### Stage 4 and 5: Data extraction, analysis, and report

Data was extracted independently by two reviewers using a data extraction table agreed by all team members **(**S2 Appendix**)** while any disparity was discussed and compared to agree on final data extraction. The data extraction table was customised to ensure extraction of important data including:

- Article identifier: designated number; title; author
- Article epidemiology: year; country
- Study sample: details of study population, including model e.g. assay (name of assay), *in vitro* (types of cell lines), *in vivo* (type of animal model, number of animals), and clinical (type of study: randomised controlled trials. cohort, qualitative, observational, others; characteristic of population studied: disease, healthy, number of patients, gender, age)
- Outcomes: pharmacological (e.g. anti-inflammatory), disease (e.g. diabetes) and physiological (e.g. central nervous system) categories of outcomes studied (based on individual study objectives, to aid in identifying research gaps); summary of important findings
- Reasons for exclusion must be stated in remarks (e.g. not hemp specific, non-primary paper, not related to study objectives)

Analysis to provide descriptive numerical summary of hemp research trends based on number and type of publication per year, country, disease categories, and pharmacological properties was conducted and presented in charts and figures. Research gaps were also identified based on level, number, type of evidence, and categorical outcomes reported for each individual disease and pharmacological property, and as an overall for all included articles. The research gaps for individual disease category and pharmacological effects were presented numerically and descriptively in a table. In line with scoping reviews concept and methods, in addition to the non-uniformity of test item selection used as intervention, quantitative assessment of included articles’ quality was not carried out. The Preferred Reporting Items for Systematic Reviews and Meta-Analyses extension for scoping review (PRISMA-ScR) checklist [23] was used to guide the reporting of this scoping review (S1 Checklist).

## Results and discussion

### Studies inclusion

**Fig 1:**
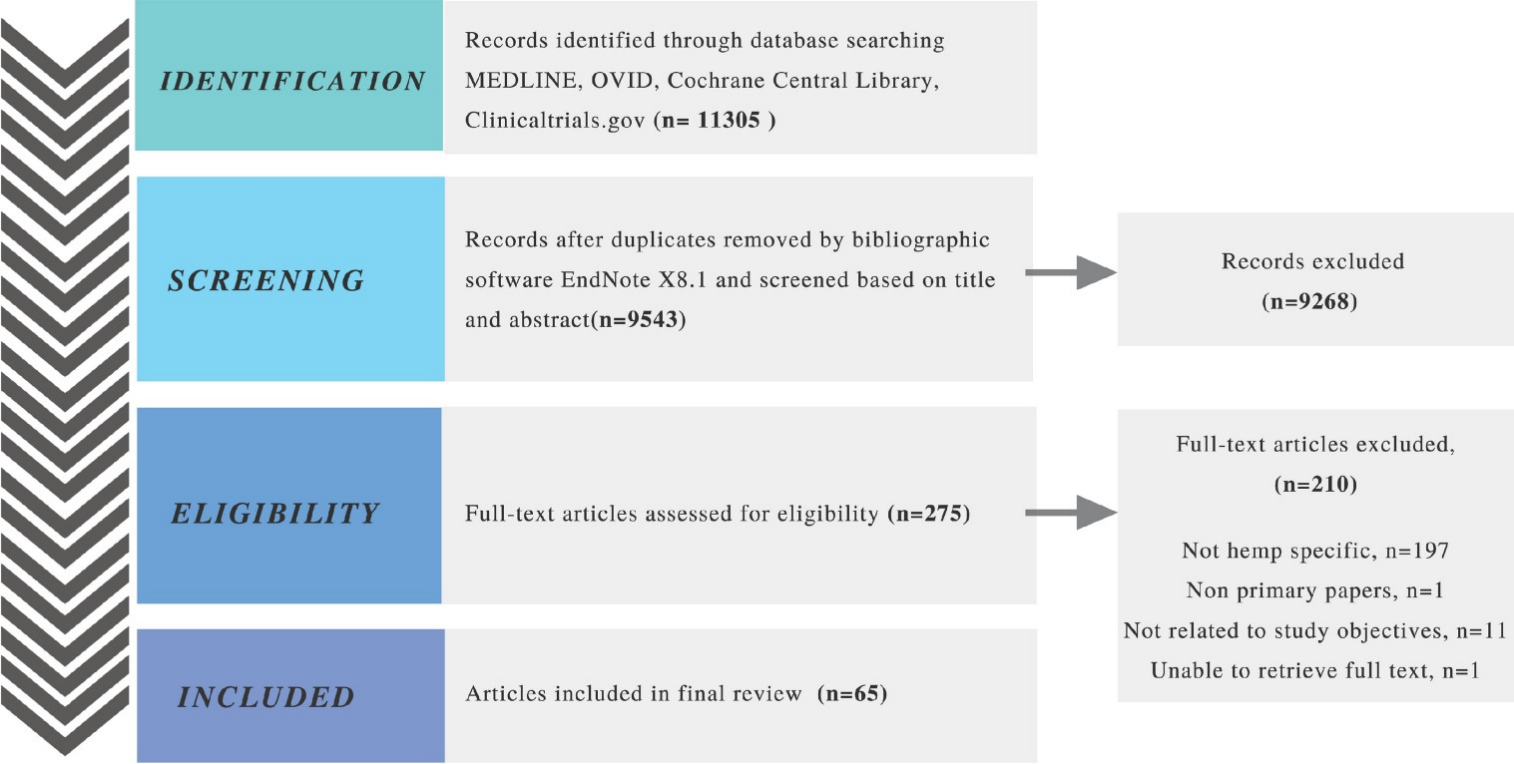
Preferred reporting items for systematic reviews and meta-analyses (PRISMA) flow diagram of screening process.

A total of 11305 records were initially identified after a keyword search. Following title, abstract, and full text screening according to inclusion and exclusion criteria, a final 65 articles were included for descriptive and numerical analysis in this scoping review, as outlined in the PRISMA flow diagram (Fig 1). The relatively small number of article included compared to the results on initial research suggests that hemp research only make up a small proportion of the total pool of cannabis-related research, similar to marketed cannabis-based drug trends which are either derived from marijuana or synthetically produced [11, 24].

During search and screen, we found it challenging to identify articles that clearly distinguish between hemp and marijuana. Search engines of the electronic databases used were unable to separate hemp-specific papers from papers that reported on marijuana and cannabis in general even though we had applied the search keyword “hemp” only. In addition, search on the MEDLINE database also resulted in papers of both agricultural weed and medical/recreational weed (i.e. marijuana). All these contributed towards the large number of non-hemp-specific articles excluded.

### Hemp research scientific evidence: Topics, trends, and gaps

**Fig 2:**
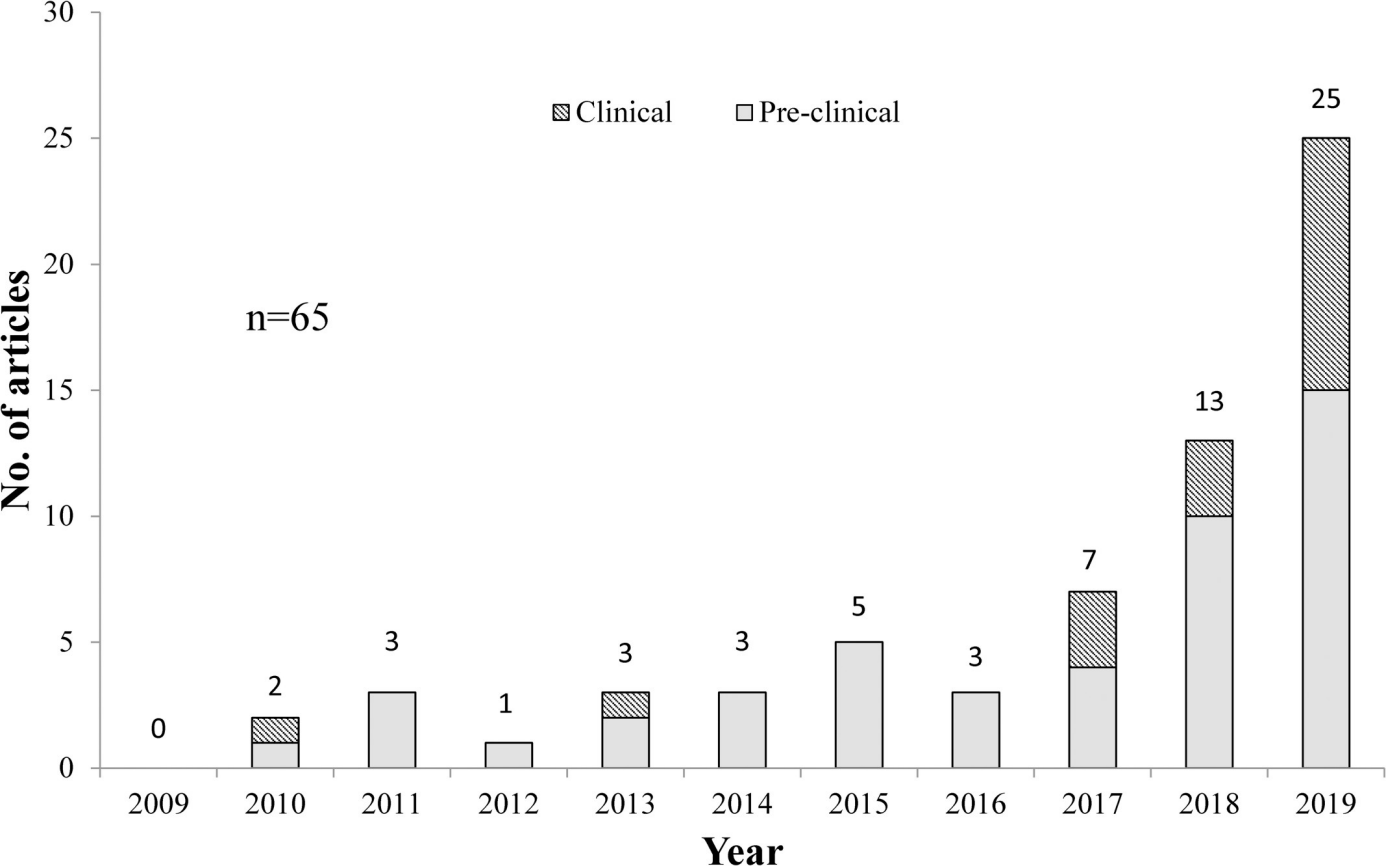
Number of hemp-specific primary articles published from year 2009–2019.

No hemp-specific primary articles were published in 2009. From year 2010 to 2017, few hemp-related studies were published with a steady rise in numbers. There is an evident surge from year 2018 onwards, representing growth of interest in this field of study, which is more than double of 2017’s total. In 2019, the number of clinical papers exceeded threefold the amount of clinical papers published in 2018 (Fig 2). This sudden increase in hemp research may be attributable to the easy accessibility of marketed hemp products, following the U.S. 2018 Farm Bill removing hemp from the Controlled Substances Act [25].

**Fig 3:**
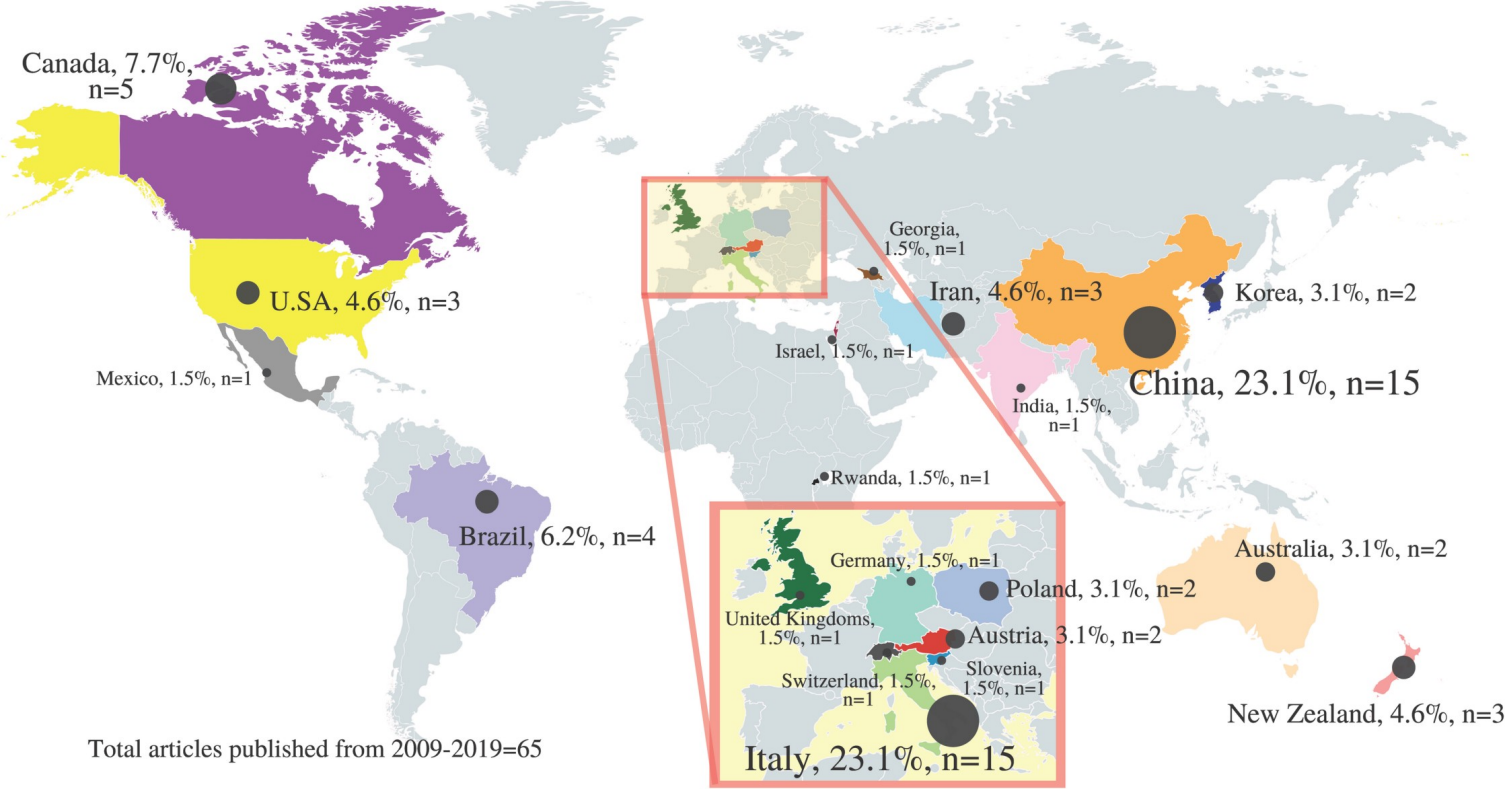
Country distribution of hemp-specific primary articles published from year 2009–2019. (Adapted and reprinted from www.mapchart.net/world.html under a CC BY license, with permission from mapchart.net, original copyright 2014).

Fig 3 shows the country distribution of hemp-specific primary articles published from year 2009 to 2019. China and Italy led the hemp research field with the highest number of hemp-related primary research articles (n = 30/65; 46.2%), accounting for almost half of the total. In descending sequence, from most to least number of articles published by country, is China, Italy, Canada, Brazil, Iran, New Zealand, U.S.A, Australia, Austria, Korea, Poland, Rwanda, Georgia, Germany, Israel, Mexico, India, Slovenia, United Kingdoms, and Switzerland.

As China has been using hempseed in Traditional Chinese Medicine practice for a long time [26], it is not surprising that China was among the top leading countries in hemp research. Italy was among one of the first countries that legalise cannabis for medicinal use back in 2007 [27], which therefore may have contributed towards its high number of hemp biomedical research.

All studies included are of heterogeneous design and not directly comparable. Disease and pharmacological effects investigated are numerically and descriptively presented in Table 3, with their respective level and corresponding quantity of evidence and research gap identified. Among the identified hemp-specific studies, a majority were pre-clinical (n = 47/65; 72.3%), compared to clinical studies (n = 18/47; 38.3%). Overall, hempseed (n = 32/47; 68.2%) is the most common part of the hemp plant used in pre-clinical research. Only some pre-clinical studies focused on pure CBD (n = 7/37; 18.9%) as a potential active compound identified or purified from the plant, while half of the clinical studies published utilised CBD or CBD enriched hemp oil (n = **9**/18, 50%) as intervention. A huge variation, some unspecified, source of hemp cultivar was used in addition to the different formulations of hemp and CBD utilised in most studies, which made direct comparison between studies not possible. Details of all included articles can be found in Supporting Information (S1 and S2 Tables).

**Table 3:** Research gaps identified.

Main topic area	Number of studies, n		Research gaps identified
	Clinical: Level of evidence; I = RCT, II = Cohort, III = Uncontrolled prospective interventional*, IV = Cross sectional, V = Anecdotal, VI = Case Series/Report (n)	Pre-Clinical: Level of evidence; I = *in vivo*, II = in vitro**(n)	
***Disease***			
Multiple sclerosis		I **(3)** [28–30]	Extrapolation to clinical models, unclear mechanism
Epilepsy (Refractory, developmental, encephalitic)	II **(1)** [31], III **(2)** [32, 33], IV **(1)** [34]		Need for high quality evidence, unclear mechanism
Skin disorders (psoriasis, atopic dermatitis, scars, acne)	V **(1)** [35]	II **(1)** [36]	Unclear contribution of hemp in mixture formulation towards effects, herb-herb interaction; preliminary data for anti-acne effects
Alzheimer's disease		I **(1)** [37], II **(3)** [38–40]	Pre-clinical data, extrapolation to *in vivo* and clinical models
Arthritis		II **(2)** [41, 42]	Extrapolation to *in vivo* and clinical models
Cancer	VI **(2)** [43, 44]	I **(1)** [45], II**(2)** [46, 47]	Low level of evidence, translation to *in vivo* and clinical models, differential anti- and pro-proliferative effects in cells
Constipation	I **(3)** [26, 48, 49]	II **(1)** [50]	Unclear contribution of hemp in mixture formulation towards effects, herb-herb interaction
Cannabis dependence	III **(2)** [51, 52]		Need for high quality evidence
Hypercholesterolaemia	III **(1)** [53]	I **(2)** [37, 47], II **(4)** [37, 47, 54, 55]	Extrapolation to *in vivo* and clinical models
Dysautonomic syndrome	III **(1)** [56]		Low level of evidence, unclear mechanism
Fragile X Syndrome	VI **(1)** [57]		Low level of evidence, unclear mechanism
Hypertension		I **(2)** [58, 59], II **(1)** [17]	Extrapolation to *in vivo* and clinical models, unclear mechanism
Diabetes and related complications		II **(3)** [60–62]	Extrapolation to *in vivo* and clinical models
Arthritis		II **(2)** [41, 42]	Extrapolation to *in vivo* and clinical models
Menopause		I **(1)** [63]	Extrapolation to clinical models, unclear mechanism
***Pharmacological Effects***			
Anxiolytic	I **(1)** [64]		Single anxiety model used (public speaking), U-shaped dose response
Neuroprotection (including anti-neuroinflammatory)		I **(5)** [28, 29, 65–67], II **(3)** [16, 68, 69]	Extrapolation to *in vivo* and clinical models
Anti-inflammatory (excluding anti-neuroinflammatory)		I **(1)** [70], II **(6)** [16, 36, 71–74]	Extrapolation to *in vivo* and clinical models
Analgesic	II **(1)** [75]	I **(1)** [76]	Extrapolation to *in vivo* and clinical models, unclear mechanism
Myorelaxant	I **(1)** [77]		Need for high quality evidence, unclear dose and contribution of hemp towards effects
Antioxidant		I **(4)** [37, 65, 71, 78], II **(12)** [39, 40, 46, 73, 79–86]	Extrapolation n to *in vivo* and clinical models
Antimicrobial		II **(3)** [36, 39, 87]	Extrapolation to *in vivo* and clinical models, resistance potential
Renal protection		I **(1)** [88]	Extrapolation to *in vivo* and clinical models
In reproductive system		I **(2)** [78, 89]	Toxicology study of various formulations and duration

**including assay studies.

*including pragmatic trial, pilot RCT, non-randomised and non-blinded interventional studies.

Despite popular claims of health benefits on hemp on various websites from a basic google search performed using the search phrase “health benefits of hemp”, we found no strong evidence to support the use of hemp for any specific indication. Here, we selectively discuss a few popular claims of hemp’s beneficial effects promoted to the public on commercial websites and scientific evidence that are interesting to our team of investigators.

#### Multiple sclerosis

Multiple sclerosis is a disabling autoimmune disease involving nerve degeneration and currently there are few reliably curative treatments [90]. Currently, a marijuana based oromucosal spray (Sativex), containing THC:CBD at 1:1 ratio is available as add-on treatment for spasticity among multiple sclerosis patients, supported by several RCTs and reviews [91–93]. The proposed mechanism of action for Sativex is unclear, though it is thought that CBD and THC synergistically act on non-cannabinoid receptors to exert its therapeutic effects [94].

In animal studies of experimental autoimmune encephalomyelitis, an early stage multiple sclerosis model, hemp-derived CBD and hempseed oil in combination with evening primrose oil showed some improvement of general clinical disease scores and inflammatory markers [28–30]. Among the potential mechanisms discovered are CBD’s anti-inflammatory actions in reducing CD4 and CD8α T-cell release, activating astrocytes, and increasing anti-inflammatory cytokine interleukin (IL)-10 in the spinal cord of affected rats [28]. Other potential mechanisms of CBD that may collectively contribute towards neuroprotection *in vivo* include anti-apoptotic and anti-oxidative effects [28, 29]. Whole hempseed oil also demonstrated anti-inflammatory activities via heightened expression of anti-inflammatory IL-10 and Rapamycin-insensitive companion of mammalian target of rapamycin (RICTOR) genes with beneficial effects towards myelin survival in experimental autoimmune encephalomyelitis [30].

Although both marijuana and hemp belong to the same plant species *C*. *sativa*, no clinical papers were identified to support hemp’s therapeutic use in multiple sclerosis. In the meantime, available data on CBD’s ability to target various neuro-inflammatory pathways *in vivo* in pre-clinical studies are valuable [28, 29]. Exploring the potential benefits of other bioactive compounds of hemp beyond CBD may also be useful.

#### Epilepsy

Another marijuana-based oral solution, Epidiolex, contains CBD and has been approved for treatment of rare and severe forms of epilepsy including Lennox-Gastaut syndrome and Dravet syndrome [10]. One prospective cohort study on CBD derived from hemp suggested some potential benefits in improving seizure control in refractory epilepsy. However, evidence of efficacy is insufficient due to the high proportion of non-responders [31]. Another retrospective cross-sectional survey investigating the use of various hemp oil formulations including those containing less than 0.1% THC; as well as other formulations containing various THC and CBD concentrations also reported fewer seizure occurrences among 81.3% of the 53 respondents with refractory epilepsy [34]. One observational study reported that hemp-based CBD in medium chain triglyceride solution improved seizures in 37.9% of children and adolescents with developmental and epileptic encephalopathy while 26.9% of patients did not respond to treatment [33]. Lastly, an open-labelled single arm study reported the potential use of an improved formulation of highly purified CBD from hemp embedded in seamless gelatine matrix beadlets for paediatric refractory epilepsy [32]. Due to study design limitations of small sample size, inclusion of various types of epilepsies, lack of control/comparator group, lack of randomisation and blinding, self-reported retrospective study design, and a lack of statistical power for results interpretation in these studies, it is not possible to draw strong conclusions from our findings. It is also clear that more studies are needed before claims of hemp’s benefits in refractory epilepsies could be made.

#### Anxiolytic effects

Many websites promote hempseed’s benefit in soothing stress and aiding in sleep [95–97]. To the best of our knowledge, there have been no studies in humans or animals that report hempseed’s benefits in improving sleep or as an anxiolytic. However, one study did show that single-dose oral CBD from hemp exhibited inverted bell curve dose-response anxiolytic effects among 60 healthy volunteers. In this study, no sample size calculation was conducted and this reduces the statistical power of the results obtained [64]. This study was of short duration, a limitation that is also presents in another review published on CBD and anxiety disorders. This highlights the need for studies of longer duration [98]. Given that hempseed oil of different sources and cultivar contain a variety of cannabinoids including CBD at different quantities [99], it is therefore not suitable to directly imply that hemp exhibits beneficial anxiolytic effects as reported for CBD.

#### Promotion of skin health

Hemp has also been o largely promoted to benefit skin health. A retrospective, single arm, non-randomised, small sample sized study reported benefits of a commercially purchased hemp-based ointment in improving skin hydration, elasticity, and scar appearances among a largely heterogeneous group of patients with inflammatory skin diseases and scars. Due to these study design limitations in addition to the unclear content of formulation used (containing hemp and unspecified concentrations of other herbs), no significant conclusion can be drawn [35].

Hemp has shown some potential in cell studies for acne. In *Proprionibacterium acnes* infected keratinocytes, hexane extracts of hempseed were shown to inhibit 5-lipooxygenase and reduce inflammatory cytokines at low concentrations of 0.3% and to have antimicrobial effects (against *P*. *acnes*) at high concentrations (20%) *in vitro* [36]. Future studies on formulation and dose-response in *in vivo* models of different acne severity will further strengthen these findings of hempseed as an effective anti-acne agent in humans. The safety of high doses required for antimicrobial effect should also be investigated.

#### Promotion of brain health

Despite online claims of hempseed consumption promoting brain health, hemp’s effect in the central nervous system has been demonstrated only in non-human, pre-clinical studies in cells and animals as anti-neuroinflammatory, acetylcholinesterase (AchE) inhibiting, and anti-depressant effects in animal models of Alzheimer’s disease and traumatic brain injury.

*Anti-neuroinflammation*. Neuroinflammation has been reported to contribute towards many neuronal injuries, diseases, and aging, though the pathogenesis of these conditions are often multifactorial and are yet to be fully elucidated [100, 101].The anti-neuroinflammatory effects of hempseed phenylpropionamides were demonstrated *in vivo* (mice) and *in vitro* (microglia) in lipopolysaccharide-stimulated neuroinflammation models. Among the mechanisms elucidated include attenuation of pro-inflammatory cytokines such as tumour necrosis factor (TNF)-α (lignanamide compounds, Cannabisin F, Grossomide), IL-6 (Cannabisin F, Grossomide), and modulation of inflammatory pathways such as nuclear factor kappa-light-chain-enhancer of activated B cell (NF-κB) phosphorylation (Cannabisin F, Grossomide) [16, 66, 68, 69].

In an *in vivo* experimental mouse model of neuroinflammation induced by lipopolysaccharide (LPS), a phenylpropionamide extract containing at least 14 identified compounds improved memory dysfunction associated with neuroinflammation in a dose-dependent manner. Interestingly, this dose-dependency was not reflected by changes in pro-inflammatory cytokine levels of IL-1β, TNF-α and IL-6, where a higher dose of 2g/kg fared worse than the lower 1g/kg dose [66].

High dose ethanol extract (90%) of hempseed at 400mg/kg given via intragastric injection to an aging rat model induced by D-galactose for 14 weeks improved aging-related memory loss compared to normal saline. The treated group had increased superoxide dismutase levels in addition to normalised astrocyte levels, phorphorylated tau levels, and presenilin 1 expression in their brain, comparable to control rats [65].

Future investigations should focus on compounds that are synergistically targeting the multifactorial pathogenesis pathways of neuronal injuries such as inflammation, oxidative stress, neurogenesis and excitotoxicity, all of which are yet to be fully elucidated [5, 100, 101].

*Acetylcholinesterase (AChE) inhibition and Alzheimer’s disease*. AChE inhibitors are currently used for several indications including dementia and Alzheimer’s disease, as low levels of the neurotransmitter acetylcholine is one of the important pathogenesis factors thought to contribute towards progression of this disease [102]. Fragments of 6-glucosyl luteolin, 8-glucosyl luteolin, linolenic acid, linoleic acid, and oleic acid found in hemp demonstrated AChE inhibitory properties [39]. Lignanamides including Cannabisin A, Cannabisin D, and a novel lignanamide named 3,3’-demethyl-heliotropamide were also identified with acetylcholinesterase inhibition potential when compared with galantamine [40]. Hempseed protein hydrolysate fraction containing majority low molecular weight peptides of three to eight amino acids was also found to inhibit human and eel acetylcholinesterase activity in a dose-dependent manner. A kinetics study further suggests that these hydrolysates inhibit the enzyme non-competitively [38].

In another *in vivo* study, compared to control media, hempseed meal reduced the incidence of severe eye degeneration in a *Drosophilia* Alzheimer’s Disease model with Aβ42 induced cytotoxicity. However, this was not statistically tested. Linoleic acid and linolenic acid were hypothesised as possible protective agents against eye degeneration. The beneficial protective effects were not observed for Parkinson’s and Huntington’s disease model in this same pre-clinical study [37].

Current data for hemp-specific bioactive compounds with AChE inhibitory and anti-dementia activity are preliminary and therefore require further investigation. The pharmacokinetics of these agents including duration of action, ability to cross the blood brain barrier, and inhibition reversibility should be explored as accumulation of acetylcholine can be fatal [103, 104].

*Antidepressant effects*. We only found one article which investigated hemp derived CBD in a depression model of diabetic rats. Intraperitoneal CBD injection demonstrated a U-shaped dose response antidepressant effect via a forced swimming test, with an optimal dose of 30mg/kg. However, as control rats also exhibited some degree of depressive like behaviour, further studies are needed to validate these findings [62].

*Traumatic brain injury*. Traumatic brain injury is an acquired brain injury which can lead to permanent disabling effects, with no treatment [105]. In an experimental mild traumatic brain injury mouse model, oral CBD in hempseed oil compared to hempseed oil alone significantly improved some behavioural outcomes, including aggression and impaired social activities. No difference was observed in biochemical markers of excitotoxicity at 60 days post injury [67]. There was no actual comparator control group (e.g. saline, oil) without any active ingredients in this study which calls for further investigation using a better study design. Given the high failure rates of clinical trials in traumatic brain injury, well designed pre-clinical studies are much needed to improve translation to human use [106].

In summary for effects in central nervous system, there is insufficient pre-clinical evidence on the efficacy of hemp in many *in vivo* neurological models overall, though there is some potential shown by *in vitro* studies. With limited therapeutic options and high failure rates of clinical trials for such neurological disorders [106–108], more robust pre-clinical studies are needed to materialise translation into successful clinical trials.

#### Anti-inflammatory effects

It is now believed that a delicate balance between pro- and anti-inflammatory mechanisms in the body is required to promote recovery and maintain function. Inflammation is required in early stages as the body defence system against foreign insults such as infection or injury. However, prolonged and uncontrolled inflammation can lead to unwanted effects such as cell death and tissue remodelling which may further contribute to negative physiological effects such as worsened disease progression and pain [109–111]. Hemp has demonstrated anti-inflammatory activities in several cell and animal studies.

10% hempseed diet was able to reduce Cyclooxygenase-2 (COX-2) levels in hypercholesteraemic rats [71] while CBD purified from hemp demonstrated anti-inflammatory activities in a gastrochisis rat model [70]. In human polymorphonuclear cells, standardised extract containing 5% CBD, <0.2% THC and 95% cannabis vegetal complex matrix demonstrated dose-dependent anti-inflammatory effects via suppression of polymorphonuclear leukocyte migration, reactive oxygen species (ROS) generation and TNF-α production [72]. In a different study, a standardised ethanolic extract of hemp flowers (5% CBD, <0.2% THC, medium chain triglycerides, and other unspecified active compounds) suppressed TNF-α and NF-κB transcription, and reduced the release of IL-8 and Matrix metallopeptidase (MMP)-9 in human keratinocytes and fibroblasts cell lines [74]. In both studies, standardised extracts comprised of a mixture of compounds exhibited more potent anti-inflammatory properties than CBD alone [72, 74], highlighting that interaction between various compounds may contribute towards synergism [81].

Differential pro- and anti-inflammatory effects were observed in CBD-containing liquid aerosols. The tested product was pro-inflammatory in normal non-inflammatory human epithelial cells, monocytes, and fibroblasts but anti-inflammatory in lipopolysaccharide-stimulated monocytes *in vitro* [73].

From the current evidence involving *in vitro* and *in vivo* studies, the inflammatory effect differs in different inflammatory environment and cell lines. The mechanism behind these differential effects still remains unclear. The use of different formulations and test items further made direct comparison between studies difficult. More studies are required to translate such data into clinically significant models.

#### Pain

There is long standing interest in medical cannabis for pain, but mainly focusing on marijuana. There is some evidence that suggested use of medical marijuana and cannabinoids in chronic neuropathic pain that has failed other conventional therapies [112]. Unlike for marijuana, currently, there are currently limited number of high quality human trials for the use of hemp in chronic pain [75] while an animal study demonstrated anti-allodynic effects potentially via activation of 5HT1A receptors of the serotogenic system [76].

One study reported myorelaxant effects with significant reduction in pain scores among 30 patients with temporomandibular disorders who were administered CBD containing hemp-based cholesterol ointment, compared to controls. Small sample size and unspecified amount of dose administered are among the important limitations of this study [77].

Although there is currently no study to identify the bioactive compounds in hemp that can potentially contribute towards analgesic effects, future investigations exploring the synergistic effects of hemp plant or compounds with anti-inflammatory [36, 70–74] and anti-oxidative [17, 37, 39, 40, 46, 65, 71, 73, 78–86] properties to target the multimodal pain pathogenesis will be useful.

#### Cancer

Investigating new anticancer agents has always been one of the main areas of interest in biomedical research. There are some pre-clinical data demonstrating anticancer potential of hemp. *In vitro*, 80% methanol aqueous hempseed oil extract has anti-proliferative effects in human colorectal adenocarcinoma cell lines while hempseed and hempseed flour extracts show pro-proliferative effects at concentrations of 40 and 70 μg/mL [46]. In animal studies, hemp derived CBD decreased melanoma tumour size [45].

As for evidence in human use, one case report and one case series were published describing the potential effects of hemp-products containing CBD and other unidentified compounds in prolonging survival and promoting partial tumour response in brain and lung cancer [43, 44].

To date, there is no strong evidence to support use of hemp in any type of cancer as data are preliminary. The opposing pro- and anti-apoptotic effects reported in different cells are important concerns when exploring a potential anticancer agent [46]. With the current understanding that hemp of different cultivars contain a variety of compounds yet to be fully identified with various concentrations [87], further studies are needed.

#### Constipation

Hemp’s used for constipation in the form of Chinese medicine pill MaZiRenWan has been documented in several randomised controlled trials conducted by the same group of researchers [26, 48, 49]. Twice daily 7.5g MaZiRenWan capsules, containing a mixture of herbs of *Semen Cannabis Sativae* (hempseed), *Semen Pruni Armeniacae* (bitter apricot seed), *Radix Paeoniae Alba* (white peony root), *Fructus Immaturus Citri Aurantii* (immature orange fruit), *Cortex Magnoliae Officinalis* (magnolia bark), and *Radix et Rhizoma Rhei* (rhubarb root and rhizome), given for 8 weeks, was more effective than placebo in increasing the frequency of complete spontaneous bowel movement from baseline in patients with functional constipation with “excessive syndrome (overactivity of body according to Traditional Chinese Medicine)”. The benefits were maintained 8 weeks after treatment cessation. No statistical sample size calculation was done. In addition, there was a high dropout rate. Only 70% and 78.3% of participants from treatment and placebo group completed the study respectively which could contribute towards attrition bias [48, 49].

A multisite randomised controlled trial conducted across China compared oral MaZiRenWan (7.5g twice daily) with placebo and senna (15mg once daily) and showed MaZiRenWan to be comparable to senna but superior to placebo for constipation. 8 weeks after treatment cessation, patients treated with MaZiRenWan had sustained improved bowel movement, straining symptoms, quality of life measures, and colonic transit superior to both senna and placebo [26].

Active constituents that may contribute towards MaZiRenWan’s anti-constipation effect include emodin, amygdalin, albiflorin, honokiol, and naringin. These represent groups of compounds with similar structure, which significantly enhance colonic smooth muscle contraction *in vitro*. However, among all these compounds identified, none belong to hemp [50]. This observation again supports the idea of synergism of various compounds in an herbal mixture [113].

There are insufficient studies to warrant a systematic review and meta-analysis as there is only one adequately powered randomised controlled trial published to date that focused mainly on Chinese female patients [26]. Further investigation needs to be conducted to detail the mechanism of hemp contributing to such synergism.

### Safety

In the 18 clinical trials included, adverse reactions reported for CBD, hemp products and MaZiRenWan are generally tolerable with no permanent disabling effects, though these may have contributed towards drop outs in some studies (up to 30%) [26, 31, 32, 34, 35, 43, 44, 48, 49, 51–53, 56, 57, 64, 75, 77].

One study showed that hemp has negative impacts on the reproductive system of pregnant rats and their litters and therefore cautioned against its use by pregnant women [89]. There is also concern for contaminated commercially available products that could contain heavy metals not declared by marketing companies. These may have negative impact on users [73]. To objectively draw conclusions on safety and dose-dependent toxicity scientific evidence, a separate systematic or scoping review should be conducted as our search strategy was not focused solely on safety studies. A detailed analysis of the actual compounds in commercially sought products is important to determine their safety.

### Overall research gaps, limitations, and potential biasness of current available evidence

Although hemp has been investigated for a wide range of indications, there is insufficient high-quality evidence to support its use for any specific therapeutic purpose. Almost all clinical studies had major study design limitations which reduced the evidence quality. Pre-clinical studies have reported differential findings on hemp exhibiting both pro and anti-inflammatory [73], as well as anti-oxidant [37, 79–81] effects. Most importantly, overall for both clinical and pre-clinical evidence, the difference in test item selection and methods of preparation made comparison between studies challenging, even though all evidence included were categorised as hemp research.

One of the most significant pitfalls identified overall is the scarcity of quantitative data and heterogeneity in potentially bioactive cannabinoid and non-cannabinoid compound levels of test items used in both pre-clinical and clinical trials. In particular, although most papers stated the geographical origin of the hemp plant used, only a small number of articles specified the cultivars of plant utilised. Due to the versatility in hemp plant cultivation which can result in different ranges of phytochemical content, this lack of quantitative data on various potentially bioactive compounds is a significant confounding factor in a majority of the published papers. In addition, growing use of non-pharmaceutical grade hemp formulations in recent trials [35, 44, 57, 73, 77] without independent analysis of formulation content further adds to the complexity of data interpretation.

Potential contamination of the hempseed with THC (due to uncleaned hempseed) and oestrogen effective compounds from pesticide residue in some studies [39, 63] also may affect study results. Collectively, all of these limitations made it challenging to draw a robust conclusion.

### Challenges and way forward

Moving forward, although hemp’s standardisation by its THC upper limit appears sufficient to cater for industrial needs, a more stringent definition may be required to help standardise the selection of hemp for biomedical research purposes. Due to the discrepancies in other bioactive compound levels (apart from THC) among hemp plants of different origin and cultivar, it is challenging to make direct comparisons between studies for similar indications based on current available data. With the increasing ease of accessibility to hemp from various cultivars across the globe, data from future studies will be more challenging to interpret and compare if studies are continued to be conducted without identification, quantification, and standardisation of known and unknown active compounds in the hemp plant and formulation investigated. For effective herbal drug development, a pathway based strategy should be adopted covering aspects of quality control, drug discovery, and pharmacological function.

### Review limitations

There are several limitations to our review. Only English studies of the past ten years were included. Therefore, due to search duration and language limits we may have missed out older studies and studies reported in foreign language, especially studies conducted in China as the Chinese have been using hempseed traditionally for a long time. We were also unable to hand search for grey literature as Cannabis research is not conducted in Malaysia. Although attempts were made to contact lead investigators of clinical trials on hemp with pending, unclear or unpublished data, we did not receive any response.

It is clear that the sudden rise in numbers of studies occurred only after year 2017 which then surged drastically in 2019. Hence, we believe that our search duration was still able to cover the bulk of evidence. As for language restriction, it is known that there are insufficient high quality trials to support the therapeutic claims of Chinese Herbal Medicine in the past due to challenges in trial design as well as significant differences in understanding the human physiological system when compared to conventional medicine [26]. Therefore, our chances of excluding high quality Chinese language papers are low. Given the high number of articles that were still screened, we believe that our search and findings spanning the past decade is still a good indicator to sufficiently scope and produce an overview of current available scientific evidence.

Only hemp-derived CBD evidence is included in this paper. Hence our review could not draw conclusions for all CBD related evidence, which needs to take into account synthetic and CBD from marijuana. Several CBD reviews for specific therapeutic indications have been published [98, 114–118] and it was therefore not within our review aim to address this objective.

Lastly, although we presented some data on adverse reactions of hemp, it may not be an accurate representation of the full picture of hemp’s safety as we did not specifically search for toxicity studies. To address this research question, it will be more appropriate to systematically analyse toxicity and safety data in a separate review, given the bulk of data and studies that were already included in this paper.

## Conclusions

There is a growing number of studies conducted on hemp and hemp-derived compounds, representing an increasing interest in this field of research. Most hemp-specific evidence is preliminary with insufficient high quality clinical evidence for any specific therapeutic indications. In addition to cannabinoids, a few non-cannabinoid bioactive compounds including protein hydrolysates and propionamides demonstrated some pharmacological potential which can be further explored. There is limited quantitative data and uniformity in the contents of hemp extracts and formulations investigated. As hemp is highly varied in both cannabinoid and non-cannabinoid contents, there is a need for more focused selection on hemp as test items for biomedical research purposes. Improved methodological design is an important factor in strengthening and focusing future hemp research.

## Supporting information

S1 Checklist PRISMA extension for scoping review checklist. (DOCX)

S1 Appendix Search strategy. (DOCX)

S2 Appendix Data extraction table. (DOCX)

S1 Table Details of clinical studies of hemp. (DOCX)

S2 Table Details and characteristics of pre-clinical hemp-specific studies. (DOCX)
