# Supplementary material for: Cannabis sativa subsp. sativa’s pharmacological properties and health effects: A scoping review of current evidence
Source: PLoS One. 2021 Jan 19;16(1):e0245471. doi: 10.1371/journal.pone.0245471 (PMC7815160; doi:10.1371/journal.pone.0245471)
Supplement: S1 Appendix — (DOCX) [file pone.0245471.s002.docx]

**S1 Appendix**

Search strategy and keywords for: ***Medline/ Ovid/ Cochrane Library Central***

**#1:** Medicinal

**#2:** Therapeutic

**#3:** Benefit

**#4:** Effect

**#5:** Properties

**#6:** Bioactive

**#7:** #1 OR #2 OR #3 OR #4 OR #5

**#8:** Hemp

**#9:** Hemps

**#10:** Cannabidiol

**#11:** #8 OR #9 OR #10

**#12:** #7 AND #11

**#13:** Year limit 2009-2019

Search strategy and keyword for: ***Cochrane Library Central (Clinical Trials/ Clinicaltrials.gov)***

**#1:** Hemp

**#2:** Year limit 2009-2019
