# Supplementary material for: Cannabis sativa subsp. sativa’s pharmacological properties and health effects: A scoping review of current evidence
Source: PLoS One. 2021 Jan 19;16(1):e0245471. doi: 10.1371/journal.pone.0245471 (PMC7815160; doi:10.1371/journal.pone.0245471)
Supplement: S2 Appendix — (DOCX) [file pone.0245471.s003.docx]

**S2 Appendix. Data extraction table**

| **No** | **Journal, Author** | **Epidemiology** | | **Study Sample** | | | | | **Intervention** | | | | | |
| --- | --- | --- | --- | --- | --- | --- | --- | --- | --- | --- | --- | --- | --- | --- |
|  |  | **Year** | **Country** | **Study Model** | | | | | **Hemp Specific** | **Part of plant** | **Source of plant** | **Formulation** | **Bioactive compound** | **Duration** |
|  |  |  |  | **Phytochemistry** | ***In silico*** | ***In vitro*** | ***In vivo*** | **Human** |  |  |  |  |  |  |
|  |  |  |  |  |  |  |  |  |  |  |  |  |  |  |
|  |  |  |  |  |  |  |  |  |  |  |  |  |  |  |
|  |  |  |  |  |  |  |  |  |  |  |  |  |  |  |

| **Comparison** | | | | **Outcomes** | | | | **Include/**  **Exclude/**  **Unsure** | **Remarks (reason for exclusion)** |
| --- | --- | --- | --- | --- | --- | --- | --- | --- | --- |
| **Name** | **Dose** | **Formulation** | **Duration** | **Effect studied/**  **Pharmacological action** | **Disease studied** | **Physiological system studied** | **Findings** |  |  |
|  |  |  |  |  |  |  |  |  |  |
|  |  |  |  |  |  |  |  |  |  |
|  |  |  |  |  |  |  |  |  |  |
