# Supplementary material for: Cannabis sativa subsp. sativa’s pharmacological properties and health effects: A scoping review of current evidence
Source: PLoS One. 2021 Jan 19;16(1):e0245471. doi: 10.1371/journal.pone.0245471 (PMC7815160; doi:10.1371/journal.pone.0245471)
Supplement: S1 Table — (DOCX) [file pone.0245471.s004.docx]

**S1 Table. Details of clinical studies of hemp.**

**Author, Year Study type Study population Intervention, Source Comparison Health effects**

***Cheng et al.*, 2010**  Phase 1: Dose determination n=96 (32/arm) MaZiRenWan pills Placebo Increased complete spontaneous bowel;

Phase 2: Randomised Controll- n=120 (60/arm) Commercially available movement (CSBM);

ed Trial (RCT), double-blinded, (contains *Fructus Cannabis,* Decreased severity of constipation;

prospective **Population:** Adults with *Semen* *Armeniacae, Radix et* Decreased straining of evacuation;

functional constipation *Rhizome, Fructus Aurantii* Decreased use of rescue therapy

**Gender:** Female(majority) *immaturus,Cortex Magnoliae* Sustained benefits 8 weeks after treatment

and male *officanalis,Radix Paeoniae albaI* completion;

**Age:**18-65 years No difference in global and individual symptom

**Dose:** 7.5g BD assessments scores

**Duration**: 8 weeks treatment,

8 weeks follow up **Adverse events:** 11/60 patients including

flatulence, headache, abdominal pain,

passing gas, nausea

***Bian et al.,* 2013**  RCT, double blinded, n=120 (n=60/arm) MaZiRenWan pills Placebo Increased CSBM;

prospective Commercially available Decreased severity of constipation;

**Population:** Adults with (contains *Fructus Cannabis,* Decreased straining of evacuation;

functional constipation *Semen* *Armeniacae, Radix et* Decreased use of rescue therapy;

**Gender:** Unspecified *Rhizome, Fructus Aurantii* Sustained benefits 8 weeks after treatment

**Age:** 18-65 years *immaturus,Cortex Magnoliae* completion; *officanalis,Radix Paeoniae albaI* No difference in global and individual symptom

assessments scores

**Dose:** 7.5g BD **Duration**: 8 weeks treatment, **Adverse events**: 11/60 patients including

8 weeks follow up bloating, passing gas, diarrhoea, abdominal pain

***Aguirre-Velazquez et al*.,** Cross sectional survey, n=43 Cannabidiol (CBD) (from None Decreased in convulsions (81.3% );

**2017** retrospective various sources including both No change (11.6%) ;

**Population:** Mexican children hemp-specific and unspecified Exacerbation of seizure (4.6%)

with refractory epilepsy sources) alone OR in

**Gender:** Both female and combination with **Adverse events:** 16/43 patients including

male tetrahydrocannabidiol (THC) appetite changes, sleep disturbances,

**Mean age:** 7.6±4.3 years as add-on therapy gastrointestinal disturbances, increase in seizures,

(9 months-18 years) tics

**Dose:** <1mg/kg up to 9mg/kg/day,

5 cases with unknown dose

**Duration:** <1 month- 12 month

***(continued on next page)***

**Author, Year Study type Study population Intervention, Source Comparison Health effects**

***Palmieri et al*., 2017**  Uncontrolled, n=21 Commercial CBD-rich (15%) None Improved physical functionality, vitality, pain

open-labelled, prospective hemp oil (Elixinol, USA) and social role functioning;

**Population:** severe somatoform No difference in role limitations due to

and dysautonomic syndrome **Dose:** Initially 25mg/day, emotional functioning

following Human titrated up to 150mg/day then

papilloma virus (HPV) slowly reduced to 25mg/day **Adverse events:** hyperglycaemia, sleepiness,

vaccination **Duration:** 12 weeks confusion (2 patients withdrew)

**Gender:** Female

**Mean age:** 16.7 years

(12-21 years)

***Zuardi et al*., 2017** RCT, double-blinded, n=60 (n=12/arm) Commercial CBD (crystalline, Clonazepam 1mg, placebo Anxiolytic compared to placebo and clonazepam

prospective 99.6% pure), BSPG-Pharm, with an U-shaped dose response curve UK

**Population:** Healthy adults **Adverse events**: not reported; lower sedation

subjected to test of public **Dose:** 100mg, 300mg, 900mg level than clonazepam

speaking in a real situation **Duration:** Single-dose

(induced anxiety)

**Gender:** Both female and

male

**Age:** 18-35 years

***Beale et al*., 2018** Pragmatic trial, open- n=18 Commercial CBD (crystalline, None Potential restorative effects on the subicular

labelled, prospective 99.5% pure), BSPG-Pharm, and CA1 hippocampal subfields of regular

**Population:** Regular cannabis UK cannabis users, especially in heavy users

users observed for effects on

hippocampal subfield volumes **Dose:** 200mg **Adverse events**: not reported

**Gender**: Both female and **Duration:** 10 weeks

male (majority)

**Median age:** 25 years **(**18-55

Years)

***Neubauer et al*., 2018**  Cohort study, retrospective n=66 Commercially prepared CBD None Seizure improvement more than 50%= 48.5% as add-on therapy Seizure-free= 21.2%

No effect= 22.7%

**Population:** Children, Powder (crystalline, >98%

adolescents and adults with pure), Bionorica, Germany **Adverse events:** 5/66 patients including unable

refractory epilepsy to walk, nocturnal enuresis, sedation,

**Gender:** Both females and **Median therapeutic dose:** eosinophilia, yellowish skin discoloration,

males 8.3mg/kg/day (3.0-22.0 mg/ elevated liver enzymes

**Age:** 6 months- 23 years kg/day)

**Median follow-up duration:**

14.0 months (6.0-29.3 months)

***(continued on next page)***

**Author, Year Study design Study population Intervention, Source Comparison Health effects**

***Solowij et al*., 2018** Pragmatic trial, open- n=20 Commercial CBD (crystalline, None Cannabis dependence: reduced euphoria;

labelled, prospective 99.5% pure gelatine capsules), depressive-like symptoms, psychotic-like

**Population:** Frequent cannabis Trigal Pharma Ltd.,UK symptoms; improved attentional switching,

users observed for effects on memory, verbal learning; greater benefits in

psychological & behavioural **Dose:** 200mg dependant users compared to non-dependant

assessment **Duration:** 10 weeks users

**Gender**: Both females and

males (majority) **Adverse events:** no deleterious effects on

**Median age:** 25 years psychological function, well-tolerate, none-

(20.6-46.8 years) reported

***Capano et al*., 2019**  Cohort study, single-arm, n=97 Commercial CBD-rich soft None 53.3% were able to reduce opioid medication; open-labelled, prospective gels (15.7mg CBD, 0.5mg THC Significant improvement in sleep quality and

**Population:** Adults with 0.3mg cannabdivatin, 0.8mg pain intensity and interference score from

chronic pain and stable on cannabidiolic acid, 0.8mg baseline;

opioid dose for ≥2 years cannbichrome, >1% botamical No significant difference in pain disability index **Gender:** 31 males, 66 females terpene blend, Ananda and 4-iterm patient health questionnaire score

**Mean age:** 56.1 years Professional from baseline

(39.0-70.0 years) **Dose:** 15mg-60mg/day Adverse events: drowsiness, palpitations, nausea

**Duration:** 8 weeks Heartburn, dry mouth, increased night time

anxiety, disturbed sleep

***Del Bo et al*., 2019** Pilot RCT, prospective n=36 (n=18/arm) Commercial hempseed oil Control (no placebo given) No improvement in serum lipid profile;

(gelatine soft gel capsules), Improved red blood cells phospholipid fatty acid

**Population:** Children and Supplement AlfaLife, composition and index

adolescents with primary Freai Pharmaceutical Italy

hyperlipidaemia add on to diet therapy **Adverse events:** not reported

**Gender:** Both females and

males **Dose:** 3g/day

**Age:** 6-15 years **Duration:** 8 weeks

***Likar et al.,* 2019** Case series , retrospective n=9 Commercial CBD capsules Prolong survival (no statistical analysis) than

Trigal Pharma Ltd.,UK expected, mean survival 22.3 months (range

**Population:** patients with as adjuvant to maximal 7-47 months)

malignant brain tumours resection and radiochemo-

**Gender:** Both females and therapy **Adverse events:** not reported

males

**Age:** 5- 61 years **Dose:** 400mg/day

**Duration**: unspecified

***(continued on next page)***

**Author, Year Study design Study population Intervention, Source Comparison Health effects**

***Mitelpunkt et al.,* 2019** Uncontrolled, open-labelled, n=16 (5 did not complete CBD from highly purified None Reduced seizure count and monthly seizure

prospective study) C. sativa extract (>93% CBD; count from baseline

<0.2% THC) in PTL101

Population: Children with capsules (seamless gelatine **Adverse events:** sleep disturbance, insomnia,

Intractable epilepsy matrix beadlets), AiFame- somnolence, increased seizure frequency,

Gender: Both females AiLab GmBH, Switzerland restlessness, aggression, difficulty eating and

majority and males drinking

Mean age: 9.1±3.4 years **Dose:** 50mg up titrated to max

450mg/day (mean maintenance

dose= 13.6±4.2mg/kg/day)

**Duration:** 12 weeks

***Nitecka-Buchta et al.,* 2019** RCT, double-blinded, n=60 Topical cholesterol ointment Cholesterol ointment Myorelaxant effect

prospective containing 20% commercial

**Population:** Patients with Charlotte’s Hemp Extract Oil

` temporomandibular disorder in formula Olive Oil

(Charlotte’s Hemp Extract Oil

**Gender:** Both females and Cannabinoid content: 7.3%:

Males 66.97 mg CBD/mL, 0.461 mg

**Mean age:** 22.9 years CBD-A/mL, and 0.28 mg

CBD-V/mL)

**Dose:** unspecified

**Duration:** 14 days

***Palmieri et al.,* 2019** Anecdotal, open-labelled, n=20 Commercial ointment, Hemp- None Improved skin hydration, elasticity,

retrospective touch organic skin care ointment, transepidermal water loss, Psoriasis

**Population:** patients with skin Hemptouch Ltd, Novo mesto, area scoring index

disorders (psoriasis n=5, atopic Slovenia (contains unspecified Did not improve scoring atopic dermatitis

dermatitis n=5 and resulting amount of CBD seed oil, index, acne disability index

outcome scars n=10) *Mangifera Indica, Calendula*

**Gender:** females and males *officinalis, Lavendula officinalis*, **Adverse events:** no irritant or allergic

**Age:** 20-80 years Chamomile, *Amyris Balsamifera* reactions documented

and butyrospermum),

**Dose:** twice daily (quantity

unspecified)

**Duration:** 3 months

***(continued on next page)***

**Author, Year Study design Study population Intervention, Source Comparison Health effects**

***Pietrafusa et al.,* 2019** Uncontrolled, open-labelled, n=29 Crystalline CBD powder, None Improved seizure frequency in 37.9% patients;

prospective Botex Pharma, (98–99% pure) Did not improve seizure frequency in 26.9%

Population: patients with mixed into a medium chain patients (no statistical analysis)

developmental and epileptic triglyceride oil solution

encephalopathy (50–200 mg CBD/mL) **Adverse events:** somnolence, decreased appetite

Gender: Both females and diarrhea

males Mean dose: 13.62±6 mg/kg/day,

Mean age: 9.34±4.7 years initial 2.5–5 mg/kg/day

(1.9-16.3 years) uptitrated to maximum 25mg/

kg/day

Mean duration: 11.2±4.4

months

***Sulé-suso et al.,* 2019** Case report, retrospective n=1 2% w/v CBD oil, MyCBD None Partial tumour response

**Population:** Patient with lung **Dose:** two drops (0.06mL, **Adverse events:** nausea

Adenocarcinoma 1.32mg CBD) twice daily for a

**Gender:** Male week then nine drops (0.3mL,

**Age:** 81 years 6mg CBD) twice daily

**Duration:** 1 month

***Tartaglia et al.,* 2019** Case series , retrospective n=2 Patient 1: Oral paste with 18%- None Observed improvement functional wellbeing

23.5% CBD, 0.03% THC, including sleep, feeding, social avoidance,

**Population:** Patients with RSHO Blue Label, Naturewell, motor coordination, anxiety, language skills,

Fragile X Syndrome Inc., San Diego, CA; sensory procession

**Gender:** Patient 1 male; Patient 2: Hemp oil, Charlotte’s

patient 2 female Web, Everyday Advanced, CWB **Adverse events:** not reported

**Age:** *Patient 1*-3 years 6 Holdings, Inc., Denver, CO

months; *Patient 2*-22 years .

**Dose:** *Patient 1*-50mg OD; *Patient 2* -43mg OD

Duration: *Patient 1*-14 months;

*Patient 2*-2.5 year

***(continued on next page)***

**Author, Year Study design Study population Intervention, Source Comparison Health effects**

***Zhong et al.,* 2019** RCT, double-blinded, n=291 (n=97/arm) MaZiRenWan pills Placebo, Senna (15mg) **Compared to placebo:**

double-dummy, prospective Commercially available Improved complete response from baseline

**Population:** Adults with (contains *Fructus Cannabis,*

functional constipation *Semen* *Armeniacae, Radix et* **Compared** to senna:

**Gender:** Both females *Rhizome, Fructus Aurantii* No difference in improvement of complete

(majority) and males *immaturus,Cortex Magnoliae* response (≥1 CSBM/week from baseline) during

**Mean age:** 45.4±12 years *officanalis,Radix Paeoniae albaI*  treatment (primary outcome); Improved secondary outcomes of constipation

**Dose:** 7.5g BD (≥1 CSBM/week from baseline 8 weeks post

**Duration**: 8 weeks treatment, treatment follow up; colonic transit;

8 weeks follow up severity, straining, incomplete evacuation,

global constipation symptoms

**Adverse events**: bloating, mild diarrhoea,

dizziness, headache, abdominal pain, stomach

discomfort (no significant difference between

all treatment arms)

**Abbreviations:** CBD, Cannabidiol; CSBM, complete spontaneous bowel movement;THC, Tetrahydrocannabidiol
