# Supplementary material for: Cannabis sativa subsp. sativa’s pharmacological properties and health effects: A scoping review of current evidence
Source: PLoS One. 2021 Jan 19;16(1):e0245471. doi: 10.1371/journal.pone.0245471 (PMC7815160; doi:10.1371/journal.pone.0245471)
Supplement: S2 Table — (DOCX) [file pone.0245471.s005.docx]

**S2 Table. Details and characteristics of pre-clinical hemp-specific studies.**

**Author, Year Plant part used, Bioactive compound(s) Source Study type Bioactivity/ pharmacological properties**

**form**

***Saberivand et al*., 2010** Seed, oil Unspecified Unspecified *in vivo* Anti-menopausal effects

***Aukema et al.,* 2011** Seed, commercial Unspecified Commercial hemp protein *in vivo* Renal protective and cardioprotective effects in progressive

hemp protein powder (Sepallo Food Ingredients, kidney disease

Canada)

***Lee et al.,* 2011** Seed, dregs Polyunsaturated fatty acids Unspecified *in vivo* Antioxidant;

(PUFA) e.g. linoleic acid, Reduce cholesterol uptake;

linolenic acids Protective effect against Aβ42 cytotoxicity (induced

macular degeneration, Alzheimer’s Disease model)

***Yousofi et al.,* 2011** Seed, whole Unspecified Unspecified *in vivo* Negative reproductive effects on offspring;

Negative effects on lactation

***Chen et al.,* 2012** Seed, 60% ethanol extract N-trans-caffeoyltyramine, China: Bama and Yunma Phytochemical Antioxidant

Cannabisin B No. 1, two variety assay; *in vitro*

***Girgih et al.,* 2013a** Seed, commercial defatted Unspecified Commercial hempseed *in vivo* Anti-hypertensive

hempseed protein meal protein meal (Hemp Oil

Canada)

***Girgih et al.,* 2013b**  Seed, commercial defatted Unspecified Commercial hempseed Phytochemical Antioxidant

hempseed protein meal protein meal (Hemp Oil assay

Canada)

***Jeong et al.,* 2014** Seed, oil extract PUFA omega-3-fatty acids Dangjin Agriculture *in vitro* Induce rheumatoid arthritis fibroblast-like synovial cell

Technology Centre, Korea damage and death;

Anti-rheumatoid arthritis effects

***Teh et al.,* 2014** Seed, various solvent extracts Polyphenols and flavanoids Commercial hempseed cake Phytochemical Antioxidant

of defatted hempseed (Oil Seeds Extraction Ltd., assay

cake: methanol, ethanol, New Zealand)

acetone, methanol 80%,

acetone 80% a mixed solvent

of methanol: acetone: water

(MAW, 7:7:6, v/v/v)

***Teh and Birch et al.,* 2014** Seed, MAW, 7:7:6, v/v/v Polyphenols and flavanoids Commercial hempseed oil Phytochemical Antioxidant

extract of cold-pressed (Oil Seeds Extraction Ltd., assay

hemp oil New Zealand)

***(continued on next page)***

**Author, Year Plant part used, Bioactive compound(s) Source Study type Bioactivity/ pharmacological properties**

**form**

***Malomo et al.,* 2015**  Seed, commercial hemp seed protein hydrolysate Commercial hempseed Assay; Angiotensin converting enzyme (ACE) inhibitory effect;

hempseed protein meal (by alcalase, papain; or protein meal (Hemp Oil *in vivo* Anti-hypertensive

pepsin+pancreatin Canada)

***Giacoppo et al.,* 2015a** Flowerheads, dried Extracted pure Extracted CBD from *in vivo* Neuroprotective effects (improve clinical disease score and

Cannabidiol (CBD) Italian variety of industrial anti-inflammatory) in experimental autoimmune

hemp, Carmagnola encephalomyelitis with potential use in Multiple Sclerosis

***Giacoppo et al.,* 2015b** Unspecified Extracted pure CBD Extracted CBD from Italian *in vivo*  Neuroprotective effects (improve clinical disease score, anti

variety of industrial hemp, -apoptoptic, neuronal cell death) in experimental auto-

Camagnola immune encephalomyelitis with potential use in multiple

sclerosis

***Teh and Morlock,* 2015** Seed, methanol extract of 6-glucosyl luteolin or Commercial hempseed oil Assay Acetylcholinesterase (AChE) inhibitory effect;

cold-pressed hemp oil 8-glucosyl luteolin; (Oil Seeds Extraction Ltd., Antimicrobial activity against *A. fischeri*, and *B. subtilis*

linolenic, linoleic & oleic acid; New Zealand) Oestrogenic effects

4,5-dihydroxyprenyl caffeate or

caffeic acid fragments or

derivatives

chrysophanol-1-O-glucoside, Assay Antioxidant

emodin-O-glucoside and,

luteolin-7-O-glucoronide

***Yan et al.,* 2015** Seed, ethanol extract Lignanamide No.2, 7, and China: Bama county, Assay Antioxidant

9−14 Guangxi province

Lignanamide No. 7, 10, 13 AChE inhibitory effect

***Ren et al.,* 2016** Seed, freeze-dried commercial Hempseed protein peptides: Commercial hempseed Assay α-glucosidase inhibitory effect

deffated hempseed meal Leu-Arg and meal (Yunnan Industrial

Pro-Leu-Met-Leu-Pro Hempseed Company)

***Smeriglio et al.,* 2016**  Seed, cold-pressed oil Flavanoids (flavanones, Commercial hempseed of Assay Antioxidant

flavonols , isoflavones) cultivar Finola of industrial

Hemp (Scotto & D'Aulerio,

Italy)

***Teh et al.,* 2016** Seed, commercial Acid and alkali soluble Commedical hempseed cake Assay Antioxidant;

cold-pressed hempseed hemp protein isolates (Oil Seeds Extraction Ltd., ACE inhibitory effect

cake New Zealand)

***(continued on next page)***

**Author, Year Plant part used, Bioactive compound(s) Source Study type Bioactivity/ pharmacological properties**

**form**

***Aiello et al.,* 2017** Seed, deffated hempseed hempseed protein hydrolysate Industrial hemp of cultivar *in vitro*; 3-hydroxy-3-methylglutaryl coenzyme A (HMGCoA)

flour of peptin, trypsin, and Futura (Institute of Agricultural bioinformatics reductase inhibition

pancreatin;, and combination Biology and Biotechnology

National Research Council,

Italy)

***Chen et al.,* 2017a** Seed PUFA Bama county, Guangxi *in vivo* Antioxidant;

Province, China Anti-neuroinflammatory;

Improve aging-related memory loss

***Luo et al.,* 2017** Seed Grossamide Bama county, Guangxi *in vitro* Anti-neuroinflammatory

Province, China

***Zanoni et al.,* 2017** Seed, deffated hempseed hempseed protein hydrolysate Industrial hemp of cultivar Assay; HMGCoA reductase inhibition;

flour Futura (Institute of Agricultura *in vitro* Cholesterol uptake reduction Biology and Biotechnology

National Research Council,

Italy)

***Callejas et al.,* 2018**  N/A CBD Commercial CBD of hemp *in vivo* Anti-inflammatory in gastrochisis model

Origin (BSPG-Pharm, UK)

***Huang et al.,* 2018**  Seed, water extract No hemp specific bioactive Commercial MaZi RenWan *in vitro; in vivo;* Constipation relieving effects;

of commercially available compound identified (China) bioinformatics Stimulating contraction of colonic smooth muscles

MaZiRenWan (contains

*Fructus Cannabis, Semen*

*Armeniacae, Radix et*

*Rhizome, Fructus Aurantii*

*immaturus,Cortex Magnoliae*

*officanalis,Radix Paeoniae*

*alba*)

***Jin and Lee et al.,* 2018** Seed, hexane extract Unspecified Canada *in vitro* Anti-acne (anti-inflammatory, inhibit lipid synthesis, anti-

bacterial against *Propionibacterium* acnes, increase collagen synthesis, reduce *P. acnes* induced extracellular matrix damage)

***de Morais et al.,* 2018** N/A CBD Commercial CBD of hemp *in vivo* Anti-depressant effects (in diabetic model)

Origin (BSPG-Pharm, UK)

***Frassinetti et al*., 2018** Seed and sprout, 80% cannabisin A, B and C Cultivar Futura, provided by Assay; Antioxidant

ethanol extract (AssoCanapa, Carmagnola, *ex vivo* Anti-mutagenic in yeast cells (anti-cell damage)

Italy)

***(continued on next page)***

**Author, Year Plant part used, Bioactive compound(s) Source Study type Bioactivity/ pharmacological properties**

**form**

***Guo et al.,* 2018a** leaf, ethyl acetate extract HM1, HM2, HM3 Yunnan province, China Phytochemical Reversal of cholesterol transport;

16 known stilbenoids assay; *in vivo* Cytotoxicity (anti-cancer)

***Smeriglio et al.,* 2018**  Flowering top, hexane CBD, Cannabigerol (CBG), Two different chemotype *in vitro*; Aldose-reductase inhibition

Extract Cannabinol, Cannabielsoin (CBD and CBG Type), bioinformatics

β-Sitosterol, Fitol, provided by CRA-Research

Cannabichromene Centre for Industrial Crops of

Rovigo, Italy

***Zhou et al.,* 2018a**  Seed, 70% aqueous N-trans-caffeoyloctopamine Bama county, China *in vivo* Anti-neuroinflammatory, neuroprotection

ethanol extract N-trans-caffeoyltyramine

cannabisin A, cannabisin B,

N-trans-coumaroyltyramine,

N-trans-feryroyltyramine,

cannabisin C, cannabisin D,

cannabisin E, 3,3-demethyl-

grossamide, cannabisin M,

cannabisin F, isocannabisin N,

grossamide

***Zhou et al.,* 2018b**  Seed, 95% aqueous 20 compounds (mostly Bama county, China Phytochemistry; Anti-neuroinflammatory

ethanol crude extract lignanamides) *in vitro*

***Zimniewska et al.,* 2018** Stalk, 40% ethanol extract Unspecified Beniko, Wojko, Tygra, assay Antioxidant

Białobrzeskie varieties

***Alagbonsi et al.,* 2019** Seed, Leave (75%:15%), Unspecified Benin republic *in vivo*  Improve semen parameters, antioxidant

98% ethanol extract

***Belardo et al.,* 2019**  N/A CBD Commercial CBD oil *in vivo* Neuroprotection post experimental traumatic brain injury

(Enecta Group, Bologna)

***Iseppi et al,* 2019,** Inflorescences or whole Hemp essential oils (volatile Hemp service international assay Antimicrobial

plants terpenic compounds and ,France; Gift from Italy

cannabinoids) Hemp variety considered:

Antàl, Bielobrzerski, Carmag-

nola, Carmagnola CS, Dioica,

Fedora 17, Ferimon, Finola,

Futura, KC Virtus, KC Zuzana,

Markant, Santhica 27. Santhica

70, Tiborazallasi, Tygra, Zenith

***Jesus et al.,* 2019** N/A CBD Commercial CBD (BSPG *in vivo*  Antinociceptive against diabetes induced allodynia

Pharma)

***(continued on next page)***

**Author, Year Plant part used, Bioactive compound(s) Source Study type Bioactivity/ pharmacological properties**

**form**

***Kaushal et al.,* 2019** Seed, defatted hempseed Unspecified Mystique Hills, India assay, *in vivo* Anti-oxidant; anti-inflammatory; protective against

powder cardiovascular changes due to hypercholesterolaemia

***Mabou Tagne et al.,* 2019** Aerial, CM5 standardised CBD 5% and with Switzerland *in vitro* Anti-inflammatory

cannabis solvent-oil extract Tetrahydrocannabidiol (THC)

< 0.2%, and others

(unspecified)

***Malomo and Aluko et al.,* 2019** Seed, hempseed protein Hempseed protein Hempoil Canada assay AChE inhibitory effect

meal 37% pepsin hydrolysates

***Moccia et al.,* 2019** Seed, 80% aqueous methanol Unspecified Lentamente Societa Cooperativa assay, *in vitro* Pro-oxidant, antioxidant;

extract Agricola, Torrecuso BN, Italy). Pro-apoptoptic, anti-apoptoptic

Fedora hemp cv.

***Muthumalage et al.,* 2019** Unspecified Unspecified Commercial hemp formulation assay, *in vitro* Pro-oxidant, antioxidant;

(Greenroads and Hemplucid) Pro-inflammatory, antiinflammatory

***Rezapour-Firouzi et al.,* 2019** Seed, cold-pressed oil Unspecified Commercial supplement *in vivo*  Immunomodulatory effects in experimental autoimmune

(in combination with (Giah Essence Agro-Industry, encephalomyelitis

Evening Primrose Oil) &Phytopharm Company, Ira)n

***Sangiovanni et al.,* 2019** Flowers, standardised ethanolic CBD and other LINNEA Pharma *in vitro* Antiinflammatory

extract unspecified

***Simmerman et al.,* 2019** N/A CBD Commercial CBD *in vivo*  Anticancer in melanoma

(cannabidol.com)

***Wang et al.,* 2019** Seed, 75% ethanol Cannabisin F Bama county, China *in vitro*  Anti-neuroinflammatory

extract

***Winklmyar et al.,* 2019** N/A CBD Commercial CBD, *in vitro* Pro-apoptotic

(Trigal Pharma Ltd., UK)

***Wen et al.,* 2019** Seed, aether and ethanol Hempseed polysaccharide Hechi medicinal herbs market *in vitro* Antioxidant, protection against apoptosis

extract (HSP): Guangxi province, China

HSP 0: Man, Glc, Gal, and Xyl

HSP 0.2: Man, Rha, GlcUA,

Glc,Ga

**Abbreviations:** ACE, Angiotensin converting enzyme; AChE, Acetylcholineesterase; CBD, Cannabidiol; CBG, Cannabigerol; HMGCoA, 3-hydroxy-3-methylglutaryl coenzyme A; HSP, Hempseed polysaccharide; PUFA, Polyunsaturated fatty acids; THC, Tetrahydrocannabidiol
